# Cannabis: Neuropsychiatry and Its Effects on Brain and Behavior

**DOI:** 10.3390/brainsci10110834

**Published:** 2020-11-10

**Authors:** Marco Colizzi, Sagnik Bhattacharyya

**Affiliations:** 1 Section of Psychiatry, Department of Neurosciences, Biomedicine and Movement Sciences, University of Verona, 37134 Verona, Italy; 2 Department of Psychosis Studies, Institute of Psychiatry, Psychology and Neuroscience, King’s College London, London SE5 8AF, UK; sagnik.2.bhattacharyya@kcl.ac.uk

Possibly orally transmitted from before circa 2000 B.C., the first written evidence of a role of cannabis in health and disease dates back to Chinese medicine texts of the first to second century B.C. [1]. Between the 12th and the 13th century C.E., the detrimental effects of cannabis on mental health were first reported by the physician Iban Beitar [2]. Later in 1845, the French psychiatrist Jacque-Joseph Moreau described such effects as “acute psychotic reactions, generally lasting but a few hours, but occasionally as long as a week; the reaction seemed dose-related and its main features included paranoid ideation, illusions, hallucinations, delusions, depersonalization, confusion, restlessness, and excitement. There can be delirium, disorientation, and marked clouding of consciousness” [3]. Such evidence suggested a potential role of cannabis in the pathophysiology of psychosis and other mental disorders, as later corroborated by research studies performed over the last 50 years [4].

Now, in the 21st century, while the medicinal properties of cannabis are also under scrutiny through appropriate clinical development, testing and approval process, we are bombarded by claims about cannabis products that are sold over-the-counter with the promise to cure or prevent disease, improve health, and restore functioning. This has led to the question whether and why a drug like cannabis could be both a poison and an antidote. Much of the debate has been on the detrimental and potentially therapeutic effects of cannabis on brain and related behavior, with implications for a number of neuropsychiatric disorders [5].

Despite such apparent discrepancy, in recent times we have seen a considerable progress in our understanding of the role of specific cannabis ingredients and patterns of use for brain function, its neurobiology, and related behavior [6,7,8,9,10]. The chapters in this volume are but a sampling of the latest research evidence on the role of cannabis and its compounds in brain function and dysfunction as well as normal and aberrant behavior. Attention is also given to studies investigating how cannabis compounds may accelerate or prevent and even treat neuropsychiatric disorders.

Cognitive dysfunction as a consequence of cannabis use has been one of the hypotheses mostly investigated, even in this Special Issue, but also one of those mostly debated, due to conflicting evidence in both health and disease. Blest-Hopley et al. performed a systematic review of human studies investigating whether cannabis users and non-users differ in terms of memory-related brain functioning and related task performance. The authors found that cannabis use tends to be associated with poorer performance possibly underpinned by altered functioning of a wide network of brain substrates. However, they suggest that such evidence is far from unequivocal, due to difficulties in drawing conclusions from highly heterogeneous studies in terms of level and type of cannabis exposure, use during developmentally sensitive periods such as adolescence, and duration of abstinence, if any [11]. In order to clarify the effects of problematic cannabis use among young adults from both the neurophysiological and neurocognitive point of view, Imperatori et al. investigated triple-network electroencephalographic (EEG) functional connectivity in a case-control study. Results revealed an increased delta connectivity between the salience network and central executive network in the context of problematic cannabis use, specifically between the dorsal anterior cingulate cortex and right posterior parietal cortex. Such alteration, which is thought to regulate the general access to cognitive functions and to explain the development of psychopathological symptoms across multiple mental disorders, correlated with the severity of problematic cannabis use after controlling for the confounding effect of age, sex, educational level, tobacco use, problematic alcohol use, and general psychopathology [12]. In another case-control study among young adults, Shevorykin et al. investigated whether frontal alpha asymmetry (FAA), which is a measure of approach bias and inhibitory control, differs between cannabis users and healthy controls. Electroencephalographic measures revealed different patterns between the two groups, with healthy controls exhibiting greater relative right activity, that is associated with withdrawal-related tendencies, when exposed to cannabis cues during the filtering task. In contrast, cannabis users exhibited greater relative left frontal activity, which is associated with approach-related tendencies, independent of the cue. According to the authors, such a difference in using the behavioral inhibition system (BIS) and the behavioral activation system (BAS) may reflect a different organization of cognitive resources among cannabis users, with implication for emotions and behavior [13]. In another study, Sullivan et al. investigated structural brain abnormalities in the context of adolescent and adult cannabis use, finding larger cuneus surface area (SA). However, when clustering by gender, male cannabis users exhibited smaller SA and less complex local gyrification index (LGI) in frontal, cingulate and parietal regions, while female cannabis users tended to present with the opposite pattern. Moreover, independent of cannabis use, increased aerobic fitness was associated with more complex LGI and larger SA across different brain regions, possibly reflecting a superior cognitive functioning as a consequence of aerobic exercise which may mitigate the negative impact of chronic cannabis use on neurocognition [14]. Complementing this work, based on the evidence of a role of the endocannabinoid system in memory function as well as of an exercise–memory relationship, Loprinzi et al. proposed a model in which the endocannabinoid system may, at least in part, subserve the effects of exercise on memory function, through a number of endocannabinoid signaling mechanisms related to long-term potentiation, production of neurotrophic factors, and cellular neurogenesis. Its potential mechanistic paradigm, for instance, whether the site of cannabinoid receptor type 1 activation (e.g., gamma-aminobutyric acid (GABA)-ergic, glutamatergic) moderates the exercise–memory relationship, remains to be investigated [15]. Colizzi et al. discussed the importance of interpreting different lines of research evidence on cannabis and cognition altogether, including preclinical versus clinical evidence, acute versus long-term effects, occasional versus regular exposure, and organic versus synthetic cannabinoids, as a strategy to overcome the risks of interpreting the phenomenon based only on partial data. Their reappraisal concludes that earlier age of use, high-frequency and high-potency cannabis use, as well as sustained use over time and use of synthetic cannabinoids, are all correlated with a higher likelihood of developing potentially severe and persistent executive function impairments, as also corroborated by additional evidence from both structural and functional brain alterations associated with cannabis use. The authors call for attention regarding the effects that cannabis use may have in patients with neuropsychiatric conditions, whose cognitive function may already be less proficient as consequence of the underlying pathology [16].

Another recurring question in the field of cannabis and neuropsychiatry is whether the association between cannabis use and psychosis observed in many studied should be interpreted as cannabis use being a causal component in the development of psychosis [4]. Two studies published in this Special Issue advanced our understanding of the phenomenon. Colizzi et al. performed a double-blind, randomized, placebo-controlled crossover study where healthy young adults with modest previous cannabis exposure were acutely exposed to cannabis’ key psychoactive ingredient, delta-9-tetrahydrocannabinol (∆9-THC). Under such controlled experimental conditions, ∆9-THC elicited symptomatic manifestations that resembled those observed in psychosis in most of the participants, with one in five presenting with moderate to severe symptoms. Symptoms tended to quickly self-resolve; however, nearly one-third of the volunteers experienced mild symptomatic effects that lasted for at least 2.5 h [17]. van der Steur et al. performed a systematic review of the factors that may increase the risk of psychosis among cannabis users. They found that frequent cannabis use, especially on a daily basis, and the consumption of high-potency varieties, with high concentrations of ∆9-THC, are both associated with a higher risk of developing psychosis. Moreover, a common genetic background resulted to predispose to psychotic disorders as well as cannabis use, especially genetic variations in dopamine signaling. Finally, cannabis use was reported to be associated with an earlier onset of psychosis and to increase the risk of transition in individuals at clinical high risk of psychosis, thus potentially accelerating the cascade of neurobiological events leading to the manifestation of the disorder [18].

Another line of research is interested in investigating the psychobiological reasons for continuing using cannabis despite the potential experience of detrimental effects [19]. May et al. investigated the role of negative reinforcement by using the Cue Breathing fMRI paradigm which pairs a cue reactivity task with anticipation and experience of an unpleasant interoceptive stimulus, an inspiratory breathing load. Adolescents whose cannabis use reflected a substance use disorder experienced the aversive breathing load differently than experimental users and controls. However, instead of exhibiting an exaggerated activation in brain regions implicated in interoception and emotion regulation, as expected by the authors, the experience of the aversive interoceptive probe resulted in a greater deactivation across such regions. Moreover, findings did not support the hypothesis that cannabis use would be driven by negative reinforcement, as viewing substance images did not dampen uncomfortable sensations. On the contrary, results pointed in the direction of a positive reinforcement, such as increased sensation-seeking and reward responsivity, at least in adolescence [20]. A further study performed among African Americans in economically challenged areas found that current use of cannabis is more common in younger, healthier, less obese, and less educated African American older adults. In particular, findings suggest that African American older adults do not use cannabis to alleviate chronic disease, pain, or depression, and its use does not necessarily co-occur with cigarette smoking and alcohol drinking [21]. While these studies add to the increasing evidence against a self-medication hypothesis of cannabis use among both young and older people, the debate is still open.

Last but not least, cannabis use has seen a huge increase in its licit production, growing from 1.4 tons in 2000, mainly for purposes of scientific research, to 211.3 tons by 2016, due to the increasing implementation of medicinal programs with cannabis-related medicinal products for a wide range of neuropsychiatric conditions [5]. Aviram et al. reported the results of a cross-sectional questionnaire-based study aimed to investigate the impact of treatment with medical cannabis in people suffering from migraine. Medical cannabis resulted in long-term reduction of migraine frequency in >60% of treated patients, also reducing migraine disability severity and migraine analgesics consumption. Based on treatment response, indexed as a decrease in monthly migraine attacks frequency ≥50%, authors were able to identify a specific strain with potential benefits, containing higher doses of the phytocannabinoid ms_373_15c and lower doses of the phytocannabinoid ms_331_18d. As stated by the authors themselves, the anti-migraine effect of such phytocannabinoids and whether they are biological active will have to be elucidated in future studies [22]. This Special Issue also hosts the study protocol of a randomized controlled trial aiming to evaluate the efficacy of adjunctive dronabinol (licensed form of ∆9-THC) at the doses of 5 to 30 mg/die versus control (systemic analgesics only) for reducing opioid consumption in adults aged 18–65 years with traumatic injury [23].

We hope that the topics addressed in this Special Issue will result in new studies that will help further understanding the increasing role of cannabis and its components in neuropsychiatric health and disease. Thanks to such studies, we believe that in the near future we will witness important and exciting advances in the field of cannabis-related pharmacological treatments. Stay tuned.
